# Association of dietary inflammatory index, composite dietary antioxidant index and risk of death among adult cancer survivors: findings from the National Health and Nutrition Examination Survey 2001–2018

**DOI:** 10.3389/fimmu.2025.1556828

**Published:** 2025-04-07

**Authors:** Zhuanbo Luo, Shiyu Chen, Peixu Chen, Kunlong Xiong, Chao Cao

**Affiliations:** Department of Respiratory and Critical Care Medicine, Key Laboratory of Respiratory Disease of Ningbo, The First Affiliated Hospital of Ningbo University, Ningbo, Zhejiang, China

**Keywords:** dietary inflammatory index, composite dietary antioxidant index, cancer survivors, mortality, NHANES (National Health and Nutrition Examination Survey)

## Abstract

**Background:**

The development and progression of cancer can be impacted by the nutrients and components contained in the diet. This research seeks to explore the relationship between the antioxidant and pro-inflammatory properties of diet and the risk of all-cause mortality among cancer survivors.

**Methods:**

Adults aged 20 and above who had been diagnosed with cancer and participated in the National Health and Nutrition Examination Survey (NHANES) from 2001 to 2018 were selected for this study. Their survival status was verified using death certificate information from the National Death Index. The study employed two established measures, the Composite Dietary Antioxidant Index (CDAI) and the Dietary Inflammatory Index (DII), to evaluate the antioxidant and inflammatory properties of participants’ diets. A non-linear association between these two dietary indices and mortality was examined respectively using restricted cubic spline (RCS) regression. To quantify the relationship between the indices and mortality risk, multivariable Cox proportional hazards models were employed, generating hazard ratios and corresponding 95% confidence intervals. Furthermore, the study also explored the connection between the CDAI and DII.

**Results:**

In this study, a total of 3,507 cancer survivors, representing an estimated 20,016,255 cancer survivors in the US, were included in the baseline analysis. The results showed that patients with lower DII or higher CDAI values had better survival rates. RCS regression revealed that both indicators showed linear relationships with all-cause mortality in the crude and adjusted models. It was consistently noted higher CDAI or lower DII was related to a reduced risk of all-cause mortality in cancer survivors in the Cox regression. Moreover, the subgroup analysis demonstrated that these associations hold true across various subgroups, lending credibility to the overall findings of the study. At last, an inverse correlation was observed between CDAI and DII in the diets of cancer survivors.

**Conclusion:**

The research suggests that adopting a diet that low in pro-inflammatory foods and high in antioxidants may lower the all-cause mortality in cancer survivors. However, further prospective cohort studies are necessary to confirm these findings.

## Introduction

Malignant tumors represent a significant global public health issue. The global population of cancer survivors is increasing quickly, with projections indicating 1.95 million new cancer diagnoses and 600,000 deaths due to cancer in the United States in 2023 (1). Tumors are currently the second leading cause of death in many countries following cardiovascular diseases, and are expected to be the primary factor restricting increases in life expectancy during the 21st century (2).

The risk of cancer can be affected by diet in different ways, such as by changing the balance of bacteria in the gut, affecting oxidative stress levels, and managing energy intake (3). These effects are determined by the types of food consumed and whether they have properties that promote or reduce inflammation. Tools like the Dietary Inflammatory Index (DII) and Composite Dietary Antioxidant Index (CDAI) measure the inflammatory and oxidative effects of nutrients in the diet to evaluate how they influence bodily inflammation and oxidative stress. DII is calculated on the basis of 45 food parameters, including individual nutrients (such as omega-3 fatty acids), compounds (such as flavonoids), and foods (such as garlic and ginger), identified for their anti-inflammatory or pro-inflammatory properties (4). The CDAI, developed by Wright et al. (4), is a comprehensive score by measuring the intake of various antioxidants, such as selenium, zinc, and vitamins A, C, and E and selenium, zinc, carotenoid. Generally, A higher CDAI score indicates a diet rich in antioxidants, which can help mitigate oxidative stress. Diets with a high DII score and low CDAI score tend to be high in unhealthy ingredients like sugar, fat, salt, and cholesterol, leading to increased inflammation and oxidative stress. Conversely, diets with a low DII score and high CDAI score are typically characterized by high consumption of dietary fiber, vegetables, fruits, and protein, which can reduce levels of inflammation and oxidative stress. Recently, researches have consistently shown that a high DII score or a low CDAI score is linked to a higher likelihood of developing several diseases, including heart diseases, diabetes, COPD and mild to moderate chronic kidney disease (CKD) (5–8). In terms of malignant tumors, previous have investigated the impact of dietary choices on the development of tumors. Specifically, consumption of processed and red meats has been linked to an increased likelihood of gastric cancer, while a diet high fat has been associated with alterations in bile acid metabolism that could raise the risk of colon cancer (9, 10). However, limited attention has been given to the influence of diet on individuals already diagnosed with tumors. Notably, dietary interventions have the potential to ameliorate chronic inflammation, with the Mediterranean diet, characterized by plant-based foods, being a prominent example (11, 12). A recent study conducted by the National Cancer Institute has also demonstrated that a diet rich in antioxidants can enhance the efficacy of immunotherapy in melanoma patients (13).

The purpose of this research is to investigate the relationship between the antioxidant and anti-inflammatory properties of diet and mortality risk in cancer survivors, utilizing data from the National Health and Nutrition Examination Surveys (NHANES), a comprehensive and standardized nationwide study of US residents, that employed rigorous analytical methods, with the goal of shedding new light on the role of nutrition in cancer care. Assessments of diet’s antioxidant and inflammatory properties will be based on the Composite Dietary Antioxidant Index (CDAI) and Dietary Inflammatory Index (DII), two commonly used and reliable measures.

## Methods

### Study design and population

The National Health and Nutrition Examination Survey (NHANES), carried out by the Centers for Disease Control and Prevention (CDC), aims to evaluate the health status and nutritional well-being of the US population through a combination of interviews, physical examinations, and laboratory tests. The survey collects comprehensive data on population characteristics, eating habits, medical history, and biological markers. This valuable dataset serves as a critical resource for tracking health trends, pinpointing risk factors for disease, and shaping public health initiatives. The entire NHANES dataset is publicly available and can be freely downloaded from the CDC website (https://www.cdc.gov/nchs/nhanes/index.htm). The study was reviewed and approved by the National Center for Health Statistics Research Ethics Review Board, and all participants provided informed consent in writing.

**Figure 1:**
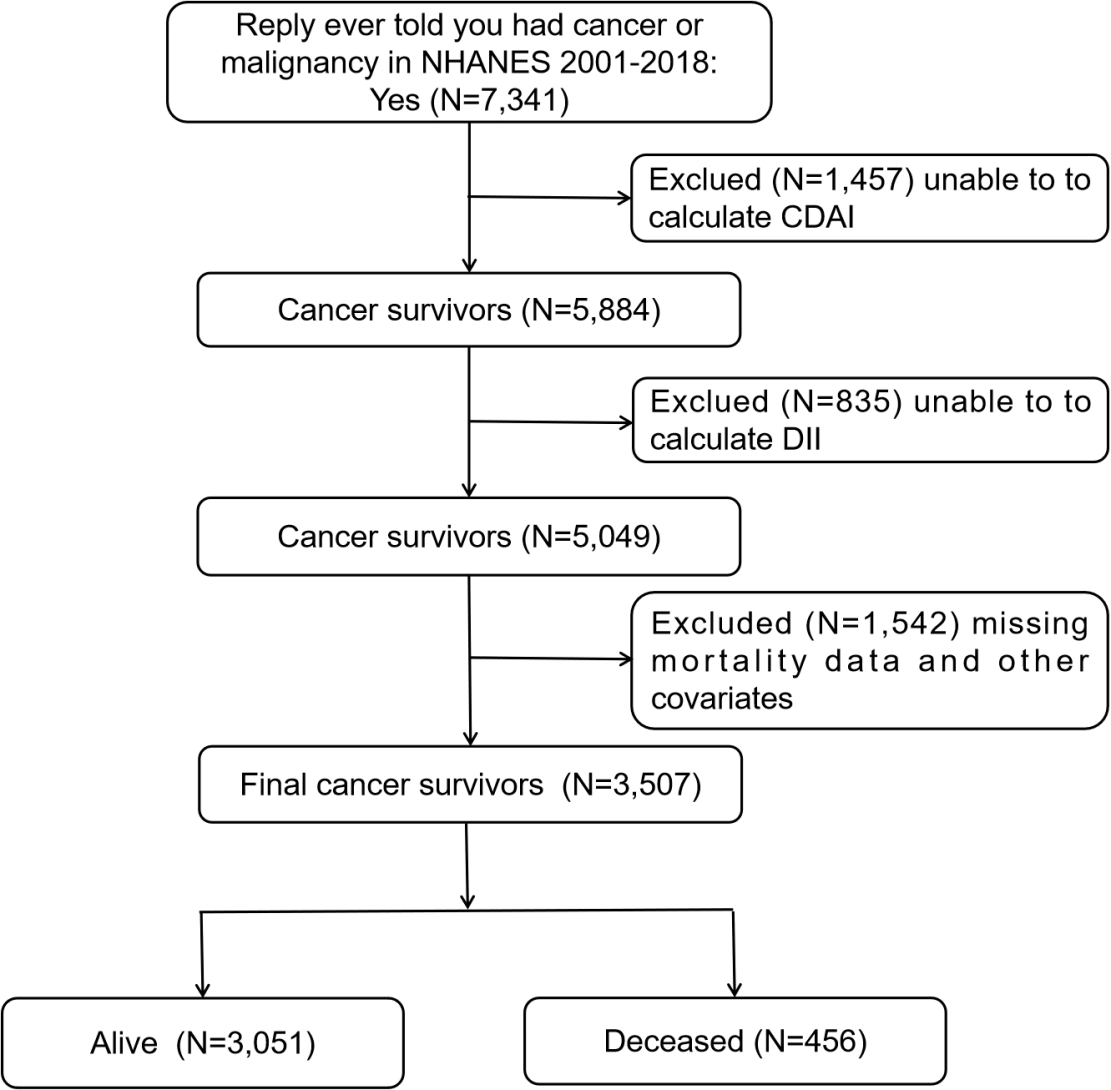
Flow chart of participants’ selection from the NHANES 2001-2018.

In the NHANES 2001-2018 survey, a total of 7,341 people completed the question “Ever told you had cancer or malignancy?” in the Medical Conditions section of the questionnaire, and replied with “Yes”. The individuals who answered “Yes” were classified as cancer survivors and included in the study. Out of the total number of cancer survivors, 1,457 participants were removed from the study because they could not compute the CDAI, while 835 participants were excluded as the DII could not be computed. An additional 1,542 participants were excluded due to missing mortality data and other confounders. Ultimately, the study included 3,507 participants, with 1,898 being female and 1,609 being male. For more detailed information, refer to **Figure 1**.

### Assessment of DII and CDAI

Data on the consumption of dietary antioxidants and other food components was collected through interviews that recalled dietary intake over two 24-hour periods. Participants were asked to provide detailed information about their food intake for two consecutive 24-hour periods, which was then used to calculate their energy, nutrient, and food component intake. The first dietary recall was collected in person during the initial NHANES visit, while the second recall was conducted over the phone 3 to 10 days later. For analysis purpose, the average estimated dietary intake of various nutrients over the two recall periods was calculated. In the absence of second recall’s data, the value for the first day was used as a substitute for the average.

The Dietary Inflammatory Index (DII) is a commonly used tool to assess dietary inflammation by examining the inflammatory effects of 45 different nutrients (14). In this study, 28 out of the 45 food parameters available in NHANES were used to determine the DII, following previously published computation protocols. To summarize, the calculation process entailed determining the Z-score for each nutrient relative to a global reference dataset (14). The Z-score was then standardized to a zero-centered distribution and scaled by the total inflammatory impact of each dietary component. Ultimately, the individual nutrient scores were aggregated to yield a comprehensive DII score. The formula for calculating DII is: (Daily intake of a dietary component - Global daily average intake of the component)/Standard deviation of the global daily intake of the component * Overall inflammatory effect score of the dietary component. This score reflects the overall impact of the diet on inflammation, with lower scores indicating more anti-inflammatory effects and higher scores suggesting stronger pro-inflammatory effects.

To evaluate the total antioxidant intake from diet, a modified version of the CDAI, developed by Wright et al., was utilized, with higher scores corresponding to greater overall antioxidant capacity (4, 15). This index was derived from average daily consumption of vitamin A, vitamin C, vitamin E, zinc, selenium, and carotenoids, as reported in two 24-hour dietary recalls. Briefly, we normalized the intake of each antioxidant(Xi) by subtracting the mean (μi) and dividing it by the standard deviation(Si); then we added the standardized intake of 6 dietary antioxidants to calculate the CDAI (15), as outlined in the formulas provided below.


$$CDAI=\sum_{i=1}^{6}\frac{X_{i}-\mu_{i}}{S_{i}}$$

### Ascertainment of mortality

In this study, we investigated the correlation between CDAI and DII and mortality rates from all causes in cancer survivors. To gather mortality data, the study linked NHANES data with the National Death Index using a probabilistic matching method that relied on personal details such as name, date of birth, social security number, and gender. The follow-up duration was calculated from the NHANES interview date until the date of death or December 31, 2019, whichever came earlier.

### Ascertainment of covariates

To minimize the impact of extraneous variables on mortality outcomes, our analysis controlled for a range of potential confounding variables. We gathered baseline data on participants through questionnaires and laboratory tests, covering factors such as age (20-40, 40-60, or >60 years old), sex (male or female), educational level (more than high school, completed high school, or less than high school), racial and ethnic background (Mexican American, non-Hispanic white, non-Hispanic black, or other race), body mass index (BMI) (<25.0, 25.0-29.9, or > 29.9kg/m^2^) and healthy eating index (HEI)-2015 (continuous). Furthermore, we assessed socioeconomic status by calculating the poverty-income ratio (PIR; the ratio of family’s income divided by poverty threshold that corresponds to the family size as defined by the US Department of Health and Human Services) and categorized it into three levels: <1.3, 1.3-3.5, and > 3.5 (16). Marital statuses were grouped into married/living with partner, widowed/divorced, or never married. Self-reported smoking habits included never smoker (smoked fewer than 100 cigarettes in their lifetime), former smoker (smoked over 100 cigarettes but no longer smoke at all), and current smoker (had smoked over 100 cigarettes in life and currently smoke) (17). Drinking status was categorized as never drinker (had less than 12 drinks in their lifetime), former drinker (had at least 12 drinks in one year but did not drink in the last year, or had not drank in the last year but had at least 12 drinks in their lifetime), current heavier drinker (consuming three or more drinks per day for females, four or more drinks per day for males, or engaging in binge drinking (four or more drinks on same occasion for females, five or more drinks on same occasion for males) on 5 or more days per month), or current mild/moderate drinker (consuming two or fewer drinks per day for females, three or fewer drinks per day for males, or engaging in binge drinking on two or fewer days per month) (18, 19).

Medical status variables taken into account included hypertension, cardiovascular disease (CVD) and diabetes mellitus (DM). Hypertension was defined as systolic blood pressure of 130 mmHg or higher, diastolic blood pressure of 80 mmHg or higher, or the use of medication to control blood pressure (6). Diabetes mellitus (DM) was diagnosed as a condition identified by a doctor or other medical provider, or glycated hemoglobin level of over 6.5%, or random blood glucose level of 11.1 mmol/L or higher, or two-hour OGTT blood glucose level of 11.1 mmol/L or higher, or the use of diabetes medication or insulin (6). Individuals who reported being diagnosed by a doctor with conditions such as coronary heart disease, heart attack, congestive failure, angina or stroke were categorized as having cardiovascular disease (6).

### Statistical analysis

Statistical analysis was conducted with consideration for the complex survey design and sampling weights of the NHANES data, ensuring that the results accurately represented the population. Continuous variables with a normal distribution were shown as mean values with standard error (SE), while those with a non-normal distribution were presented as median values with interquartile ranges. Categorical data were presented as numbers (percentages). To detect significant differences between groups, chi-square tests and one-way ANOVA were applied for categorical and continuous variables, respectively. To visualize the survival patterns linked to different classification approaches for CDAI and DII, the Kaplan-Meier method was employed. The log-rank test was then applied to assess and identify any statistically significant differences between survival curves. A restricted cubic spline (RCS) regression model with four specified knots (corresponding to the 5th, 35th, 65th, and 95th percentiles) was employed to investigate the non-linear associations between CDAI and mortality, as well as DII and mortality.

Then, both CDAI and DII were subsequently categorized into four groups based on their quartile distribution. Statistical models, including univariate and multivariable weighted Cox regression, were employed to assess the relationship between CDAI and DII scores and the risk of death from any cause among cancer survivors, with results expressed as hazard ratios (HRs) and 95% confidence intervals (CIs). Three distinct models were built: Crude Model without adjustments; Model 1 was adjusted for demographic factors such as gender, age, and ethnicity; and Model 2 further incorporated education level, family income-to-poverty ratio, smoking and drinking status, marital status, BMI, HEI2015 (in quartiles), and medical histories of hypertension, cardiovascular disease and diabetes to provide a more comprehensive analysis. Additionally, we conducted tests to assess linear trend in Cox regression models by utilizing the median value of each CDAI and DII category as a continuous variable. To investigate whether the possible effects of CDAI or DII on mortality varied across different subpopulations, a subgroup analysis was performed, dividing the entire cohort into categories based on all relevant variables.

Spearman’s correlation analysis was employed to determine the correlation coefficients among CDAI, DII, BMI, age, PIR, and HEI. A locally Weighted Scatter plot Smoothing (LOWESS) fit was applied to represent the scatter plot information of CDAI and DII. All statistical analyses were performed using the R Project for Statistical Computing (version 4.3.3), with statistical significance defined as a two-sided p-value of less than 0.05.

## Results

### Baseline characteristics

A total of 3,507 cancer survivors, representing an estimated 20,016,255 cancer survivors in the US, were included in the baseline analysis. **Table 1** displays the characteristics of the baseline samples categorized by the survival status of the cancer survivors. Compared to those in the alive group, individuals who passed away were more likely to be older males, have lower education levels and low to middle incomes, lower BMI, be widowed or divorced, have former or never consumed alcohol, be former smokers, have higher DII levels and lower CDAI levels. Additionally, those who passed away had a higher incidence of cardiovascular disease, diabetes, and hypertension compared to those who were still alive. However, there was no significant difference between the two groups in terms of HEI-2015.

**Table 1:** Characteristics of participants classified by survival status.

Variables	Total	Alive	Deceased	P value
Gender				< 0.001
Female	1898 (57.28)	1725 (60.47)	173 (46.86)	
Male	1609 (42.72)	1326 (39.53)	283 (53.14)	
Race/ethnicity				0.003
Mexican American	207 (1.93)	191 (2.19)	16 (1.08)	
Non-Hispanic black	478 (5.11)	413 (4.55)	65 (6.92)	
Non-Hispanic white	2515 (87.80)	2199 (87.53)	316 (88.68)	
Others	307 (5.17)	248 (5.73)	59 (3.32)	
Age				<0.001
20-40	225 (8.32)	221 (10.35)	4 (1.69)	
40-60	860 (34.02)	816 (40.27)	44 (13.57)	
>60	2422 (57.66)	2014 (49.37)	408 (84.74)	
Marital status				<0.001
Married/living with partner	2155 (66.95)	1904 (70.06)	251 (56.76)	
Never married	198 (5.23)	178 (5.38)	20 (4.73)	
Widowed/divorced	1154 (27.83)	969 (24.56)	185 (38.51)	
PIR				<0.001
<1.3	813 (14.79)	691 (12.34)	122 (22.80)	
1.3-3.5	1425 (35.77)	1208 (32.37)	217 (46.88)	
>3.5	1269 (49.44)	1152 (55.29)	117 (30.33)	
BMI (kg/m2)				0.01
<25	997 (28.96)	840 (27.74)	157 (32.95)	
>29.9	1284 (37.32)	1150 (38.80)	134 (32.48)	
25-29.9	1226 (33.73)	1061 (33.47)	165 (34.57)	
Education level				<0.001
Completed high school	793 (21.69)	678 (20.04)	115 (27.09)	
Less than high school	747 (12.82)	606 (9.22)	141 (24.57)	
More than high school	1967 (65.49)	1767 (70.74)	200 (48.34)	
Smoking status				<0.001
Former	1457 (39.42)	1227 (36.29)	230 (49.66)	
Never	1516 (44.52)	1349 (47.37)	167 (35.22)	
Now	534 (16.06)	475 (16.34)	59 (15.12)	
Alcohol consumption				<0.001
Current heavier drinker	331 (10.82)	306 (12.10)	25 (6.65)	
Current light/moderate drinker	1863 (59.54)	1665 (63.93)	198 (45.17)	
Former	863 (19.75)	697 (15.50)	166 (33.63)	
Never	450 (9.89)	383 (8.46)	67 (14.55)	
CVD				<0.001
No	2646 (81.30)	2361 (86.41)	285 (64.63)	
Yes	861 (18.70)	690 (13.59)	171 (35.37)	
DM				<0.001
No	2605 (78.76)	1786 (81.30)	819 (70.44)	
Yes	902 (21.24)	528 (18.70)	374 (29.56)	
Hypertension				<0.001
No	1255 (42.34)	1139 (47.44)	116 (25.68)	
Yes	2252 (57.66)	1912 (52.56)	340 (74.32)	
HEI2015	52.67 ± 0.33	52.49 ± 0.39	53.27 ± 0.51	0.21
CDAI	0.54 ± 0.10	0.67 ± 0.13	0.09 ± 0.12	<0.001
DII	1.46 ± 0.04	1.24 ± 0.05	1.73 ± 0.07	0.01

BMI, body mass index; PIR, Ratio of family income to poverty; CDAI, composite dietary antioxidant index; DII, Dietary Inflammatory Index; HEI, healthy eating index; DM, diabetes mellitus; CVD, cardiovascular disease; p value in bold indicates statistical significance.

### Association of CDAI and DII with risks of death in cancer survivors

**Figure 2:**
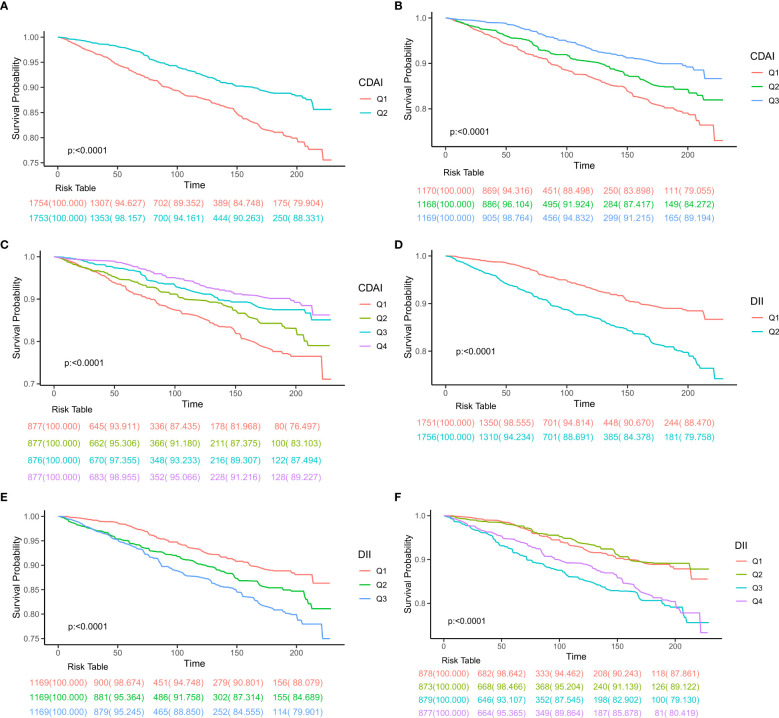
Kaplan-Meier survival plots presented for patient groups classified according to different categorization methods. **(A)** half division of CDAI, **(B)** tertile division of CDAI, **(C)** quartile division of CDAI, **(D)** half division of DII, **(E)** tertile division of DII, **(F)** quartile division of DII. CDAI, composite dietary antioxidant index; DII, Dietary Inflammatory Index.

Over an median follow-up 8.13 years, a total of 456 deaths were recorded, with 157 attributed to neoplastic reasons and 129 to cardiovascular causes, followed by chronic respiratory diseases, cerebrovascular diseases, and other factors. Participants were divided two, three, and four groups based on variations in CDAI or DII values, and Kaplan-Meier survival curves were generated. It was consistently noted that groups with lower DII or higher CDAI values had better survival rates (all log-rank test p<0.001, **Figure 2**).

**Figure 3:**
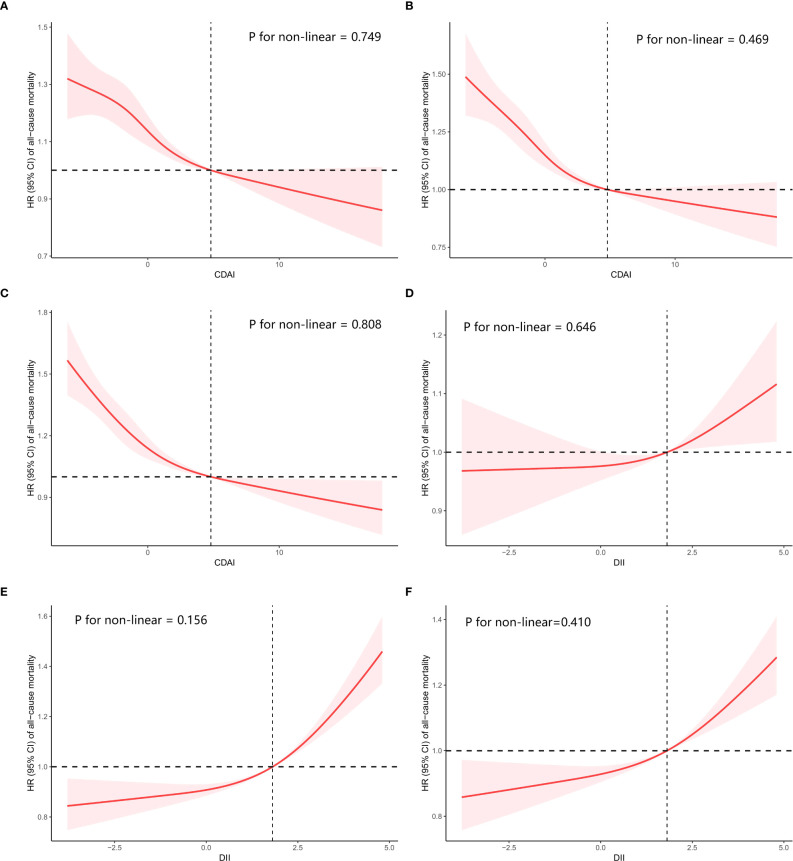
Restricted cubic spline (RCS) regression analyses the connection between two dietary indices (CDAI and DII) and the all-cause mortality in cancer survivors. **(A)** relationship between CDAI and all-cause mortality in crude model, **(B)** relationship between CDAI and all-cause mortality in model 1, **(C)** relationship between CDAI and all-cause mortality in model 2, **(D)** relationship between DII and all-cause mortality in crude model, **(E)** relationship between DII and all-cause mortality in model 1, **(F)** relationship between DII and all-cause mortality in model 2. Crude Model was unadjusted. Model 1: Adjust for age, sex and race. Model 2: Additionally adjust for education level, PIR, BMI, HEI2015, marital status, smoking status, alcohol intake, diabetes, hypertension, and coronary heart disease on the basis of Model 1.

To determine if there was a linear correlation between CDAI or DII and overall mortality, a restricted cubic spline (RCS) analysis was performed. Both indicators showed linear relationships with all-cause mortality in the crude and adjusted models (P for nonlinear>0.05, **Figure 3**).

After categorizing CDAI and DII into quartiles and using weighted Cox regression analysis, all three models showed that having a higher CDAI level or lower DII level was connected to lower likelihood of all-cause mortality. Compared to the lowest CDAI quartile, the weighted multivariate hazard ratios (HRs) for all-cause mortality were (HR, 0.84; 95% CI, 0.70-1.01) for second quartile, (HR, 0.77; 95% CI, 0.62-0.85) for third quartile, and (HR, 0.66; 95% CI, 0.62-0.77) for fourth quartile (P for trend=0.01). On the contrary, compared to the lowest DII quartile, the weighted multivariate HRs for all-cause mortality were (HR, 1.08; 95% CI, 0.89-1.30) for second quartile, (HR, 1.42; 95% CI, 1.21-2.37) for third quartile, and (HR, 2.14; 95% CI, 1.92-2.41) for fourth quartile (P for trend=0.01) (**Table 2**). Furthermore, in the continuous model, it was found that higher CDAI level or lower DII level were significantly correlated with a reduced risk of all-cause mortality in both the unadjusted and adjusted models (all p<0.05) (**Table 2**).

**Table 2:** The relationship between CDAI and DII with all-cause mortality in cancer survivors.

Variables	crude model		Model 1		Model 2	
All-cause mortality	HR (95% CI)	P	HR (95% CI)	P	HR (95% CI)	P
CDAI						
Quartile 1	ref		ref		ref	
Quartile 2	0.92(0.69,1.01)	0.07	0.71(0.60, 0.85)	<0.001	0.84(0.70, 1.01)	0.06
Quartile 3	0.84(0.66,0.92)	0.04	0.71(0.60, 0.85)	<0.001	0.77(0.62, 0.85)	0.03
Quartile 4	0.75(0.59,0.94)	0.01	0.62(0.50, 0.77)	<0.001	0.66(0.62, 0.77)	<0.001
p for trend		0.03		<0.001		0.01
Continuous	0.95 (0.90, 0.97)	<0.001	0.76 (0.63, 0.89)	<0.001	0.84 (0.82, 0.88)	0.004

	crude model		Model 1		Model 2	
All-cause mortality	HR (95% CI)	P	HR (95% CI)	P	HR (95% CI)	P
DII						
Quartile 1	ref		ref		ref	
Quartile 2	1.04(0.85,1.28)	0.70	1.16(0.97, 1.38)	0.10	1.08(0.89, 1.30)	0.44
Quartile 3	1.97(1.79,2.20)	0.01	1.26(1.03, 1.53)	0.03	1.42(1.21, 2.37)	0.01
Quartile 4	2.13(1.92,2.39)	0.03	1.62(1.34, 1.96)	<0.001	2.14(1.92, 2.41)	0.01
p for trend		0.01		<0.001		0.01
Continuous	1.42 (1.12–1.71)	<0.001	1.40 (1.22–1.63)	0.001	1.36 (1.12–1.68)	0.007

Crude Model was unadjusted. Model 1: Adjust for age, sex and race. Model 2: Additionally adjust for education level, PIR, BMI, HEI2015, marital status, smoking status, alcohol intake, diabetes, hypertension, and coronary heart disease on the basis of Model 1. HR, hazard ratio; BMI, body mass index; PIR, ratio of family income to poverty; HEI, healthy eating index. CDAI, composite dietary antioxidant index; DII, Dietary Inflammatory Index; ref, reference. P value in bold indicates statistical significance.

### Subgroup analysis and interaction

We further performed subgroup analyses to investigate the relationship between CDAI and DII with all-cause mortality in different cancer survivor groups. The subgroup analysis results consistently showed that both CDAI and DII had a significant effect on all-cause mortality, mirroring the results observed in the whole study population. Specifically, the reverse association between CDAI and all-cause mortality was observed in among individuals aged 60 years and older (HR, 0.61; 95% CI, 0.48-0.77), male (HR, 0.62; 95% CI, 0.45-0.86), non-smokers (HR, 0.57; 95% CI, 0.40-0.81) or former smokers (HR, 0.63; 95% CI, 0.47-0.85), those married/living with partner (HR, 0.69; 95% CI, 0.51-0.93), individuals with a BMI between 25 and 29.9 (HR, 0.52; 95% CI, 0.36-0.75), individuals with hypertension (HR, 0.68; 95% CI, 0.53-0.87) and DM (HR, 0.63; 95% CI, 0.44-0.90) (**Table 3**). A comparable relationship was found between higher DII level and a greater risk of death, with this correlation being particularly strong among individuals aged 20-40 years (HR, 2.42; 95% CI, 2.12-3.72) or >60 years (HR, 1.47; 95% CI, 1.20-1.79), male (HR, 1.51; 95% CI, 1.16-1.98), non-smokers (HR, 1.82; 95% CI, 1.31-2.53) or current smokers(HR, 1.69; 95% CI, 1.28-2.07), individuals with a BMI between 25 and 29.9 (HR, 1.53; 95% CI, 1.11-2.11) and those with DM (HR, 1.43; 95% CI, 1.17-2.12) (**Table 4**).

**Table 3:** Subgroup of Association Between CDAI and all-cause mortality in cancer survivors.

	Quartile 1	Quartile 2	Quartile 3	Quartile 4	p for trend	p for interaction
Age						0.21
20-40	ref	0.08 (0.01,0.78)	0.11 (0.02,0.69)	0.11 (0.01,1.05)	0.10	
40-60	ref	0.45 (0.24,0.86)	0.70 (0.38,1.28)	0.84 (0.45,1.57)	0.88	
>60	ref	0.79 (0.65,0.96)	0.76 (0.63,0.91)	0.61 (0.48,0.77)	<0.001	
Sex						0.54
Female	ref	0.83 (0.62,1.10)	0.98 (0.73,1.32)	0.76 (0.55,1.04)	0.15	
Male	ref	0.73 (0.55,0.96)	0.72 (0.55,0.95)	0.62 (0.45,0.86)	0.01	
Race						0.77
non-Hispanic white	ref	0.86 (0.70,1.06)	0.94 (0.76,1.16)	0.77 (0.60,0.99)	0.07	
non-Hispanic black	ref	0.63 (0.38,1.04)	0.65 (0.40,1.06)	0.71 (0.42,1.19)	0.26	
Mexican American	ref	1.22 (0.51,2.89)	1.22 (0.44,3.38)	0.62 (0.20,1.88)	0.32	
others	ref	0.79 (0.31,2.02)	1.27 (0.45,3.59)	0.42 (0.13,1.35)	0.33	
PIR						0.84
<1.3	ref	1.04 (0.74,1.45)	1.11 (0.73,1.70)	0.95 (0.64,1.41)	0.95	
1.3-3.5	ref	0.81 (0.59,1.10)	1.05 (0.78,1.40)	0.82 (0.55,1.22)	0.64	
>3.5	ref	1.12 (0.72,1.72)	1.04 (0.66,1.66)	0.99 (0.62,1.59)	0.85	
Smoking status						0.33
never	ref	0.73 (0.53,1.02)	0.79 (0.56,1.12)	0.57 (0.40,0.81)	0.004	
former	ref	0.77 (0.57,1.03)	0.91 (0.67,1.22)	0.63 (0.47,0.85)	0.01	
now	ref	1.06 (0.60,1.86)	1.01 (0.53,1.91)	0.92 (0.73,2.58)	0.08	
Marital status						0.34
widowed/divorced	ref	0.99 (0.71,1.38)	1.09 (0.79,1.51)	0.82 (0.57,1.18)	0.34	
married/living with partner	ref	0.80 (0.61,1.05)	0.87 (0.66,1.15)	0.69 (0.51,0.93)	0.03	
never married	ref	1.25 (0.41,3.85)	0.96 (0.32,2.92)	0.94 (0.73,2.25)	0.16	
BMI						0.08
<25	ref	1.26 (0.88,1.80)	1.47 (1.06,2.04)	0.98 (0.68,1.42)	0.86	
25-29.9	ref	0.76 (0.54,1.07)	0.72 (0.53,0.99)	0.52 (0.36,0.75)	<0.001	
>29.9	ref	0.59 (0.40,0.88)	0.70 (0.48,1.04)	0.77 (0.52,1.16)	0.33	
DM						0.11
Yes	ref	0.79 (0.56,1.11)	0.62 (0.42,0.90)	0.63 (0.44,0.90)	0.01	
No	ref	0.84 (0.65,1.10)	1.06 (0.84,1.34)	0.79 (0.60,1.04)	0.18	
CVD						0.97
Yes	ref	0.82 (0.58,1.16)	0.89 (0.62,1.28)	0.77 (0.54,1.11)	0.21	
No	ref	0.85 (0.65,1.12)	1.00 (0.78,1.28)	0.82 (0.61,1.10)	0.30	
Hypertension						0.60
Yes	ref	0.78 (0.65,0.94)	0.87 (0.72,1.06)	0.68 (0.53,0.87)	0.01	
No	ref	0.88 (0.56,1.38)	1.10 (0.76,1.60)	0.90 (0.60,1.34)	0.78	
HEI2015						0.17
Quartile 1	ref	0.72 (0.49,1.08)	0.82 (0.53,1.26)	0.46 (0.28,1.18)	0.11	
Quartile 2	ref	0.90 (0.62,1.30)	1.02 (0.68,1.53)	0.80 (0.51,1.27)	0.42	
Quartile 3	ref	0.68 (0.45,1.03)	0.65 (0.43,0.98)	0.81 (0.50,1.31)	0.60	
Quartile 4	ref	1.22 (0.74,2.02)	1.36 (0.80,2.31)	0.82 (0.49,1.37)	0.07	

BMI, body mass index; PIR, ratio of family income to poverty; HEI, healthy eating index; CDAI, composite dietary antioxidant index; DM, diabetes mellitus; CVD, cardiovascular disease; ref, reference. p value in bold indicates statistical significance.

**Table 4:** Subgroup of Association Between DII and all-cause mortality in cancer survivors.

	Quartile 1	Quartile 2	Quartile 3	Quartile 4	p for trend	p for interaction
Age						0.12
20-40	ref	1.03 (0.59, 3.77)	2.51 (0.52, 5.45)	2.42 (2.12,3.72)	0.02	
40-60	ref	0.94 (0.49,1.82)	0.71 (0.36,1.39)	1.32 (0.68,2.58)	0.55	
>60	ref	1.17 (0.97,1.41)	1.31 (1.04,1.64)	1.47 (1.20,1.79)	<0.001	
Sex						0.77
Female	ref	1.09 (0.83,1.44)	1.04 (0.74,1.45)	1.26 (0.93,1.71)	0.18	
Male	ref	1.07 (0.81,1.42)	1.11 (0.80,1.54)	1.51 (1.16,1.98)	0.01	
Race						0.76
non-Hispanic white	ref	1.01 (0.81,1.25)	0.95 (0.75,1.18)	1.10 (0.88,1.37)	0.58	
non-Hispanic black	ref	1.48 (0.47,4.70)	2.28 (0.71,7.26)	1.43 (0.43,4.78)	0.4	
Mexican American	ref	1.38 (0.80,2.37)	0.95 (0.52,1.73)	1.44 (0.76,2.71)	0.46	
others	ref	1.74 (0.59,5.13)	1.98 (0.76,5.15)	1.59 (0.49,5.12)	0.52	
PIR						0.92
<1.3	ref	0.80 (0.52,1.24)	0.71 (0.45,1.10)	1.12 (0.52,1.31)	0.49	
1.3-3.5	ref	1.02 (0.76,1.38)	0.93 (0.67,1.31)	1.84 (0.90,2.19)	0.29	
>3.5	ref	0.99 (0.71,1.39)	0.85 (0.57,1.28)	1.58 (0.65,1.80)	0.69	
Smoking status						0.13
never	ref	1.25 (0.87,1.79)	1.23 (0.86,1.77)	1.82 (1.31,2.53)	0.001	
former	ref	1.05 (0.80,1.38)	1.09 (0.81,1.48)	1.08 (0.78,1.50)	0.58	
now	ref	1.05 (0.67,1.44)	1.29 (1.11,2.23)	1.69 (1.28,2.07)	0.04	
Marital status						0.20
widowed/divorced	ref	0.92 (0.69,1.22)	0.90 (0.63,1.29)	1.00 (0.71,1.40)	0.99	
married/living with partner	ref	1.19 (0.90,1.56)	1.04 (0.77,1.40)	1.21 (0.91,1.59)	0.37	
never married	ref	0.39 (0.16,0.94)	0.28 (0.10,0.75)	1.40 (0.66,2.01)	0.09	
BMI						0.41
<25	ref	1.10 (0.78,1.56)	1.03 (0.73,1.46)	1.38 (0.69,1.59)	0.85	
25-29.9	ref	1.20 (0.84,1.72)	1.05 (0.70,1.56)	1.53 (1.11,2.11)	0.04	
>29.9	ref	0.81 (0.57,1.16)	0.88 (0.63,1.24)	1.01 (0.70,1.46)	0.83	
DM						0.06
Yes	ref	1.00 (0.71,1.41)	1.36 (0.91,2.05)	1.43 (1.17,2.12)	0.02	
No	ref	1.03 (0.80,1.33)	0.87 (0.68,1.10)	1.03 (0.84,1.27)	0.79	
CVD						0.48
Yes	ref	1.09 (0.76,1.56)	1.19 (0.82,1.74)	1.23 (0.87,1.74)	0.19	
No	ref	1.00 (0.78,1.29)	0.84 (0.66,1.09)	1.00 (0.76,1.33)	0.69	
Hypertension						0.27
Yes	ref	1.01 (0.81,1.27)	1.01 (0.80,1.27)	1.24 (0.98,1.57)	0.09	
No	ref	0.98 (0.67,1.43)	0.78 (0.54,1.14)	1.85 (1.57,2.27)	0.27	
HEI2015						0.95
Quartile 1	ref	0.98 (0.44,2.15)	1.00 (0.53,1.86)	1.20 (0.64,2.24)	0.25	
Quartile 2	ref	1.23 (0.80,1.89)	0.99 (0.64,1.54)	1.15 (0.72,1.84)	0.86	
Quartile 3	ref	1.10 (0.74,1.63)	1.21 (0.81,1.81)	1.62 (1.01,2.60)	0.07	
Quartile 4	ref	1.03 (0.74,1.43)	1.02 (0.64,1.63)	1.23 (0.58,2.60)	0.7	

BMI, body mass index; PIR, ratio of family income to poverty; HEI, healthy eating index; DII, Dietary Inflammatory Index; DM, diabetes mellitus; CVD, cardiovascular disease; ref, reference. p value in bold indicates statistical significance.

**Figure 4:**
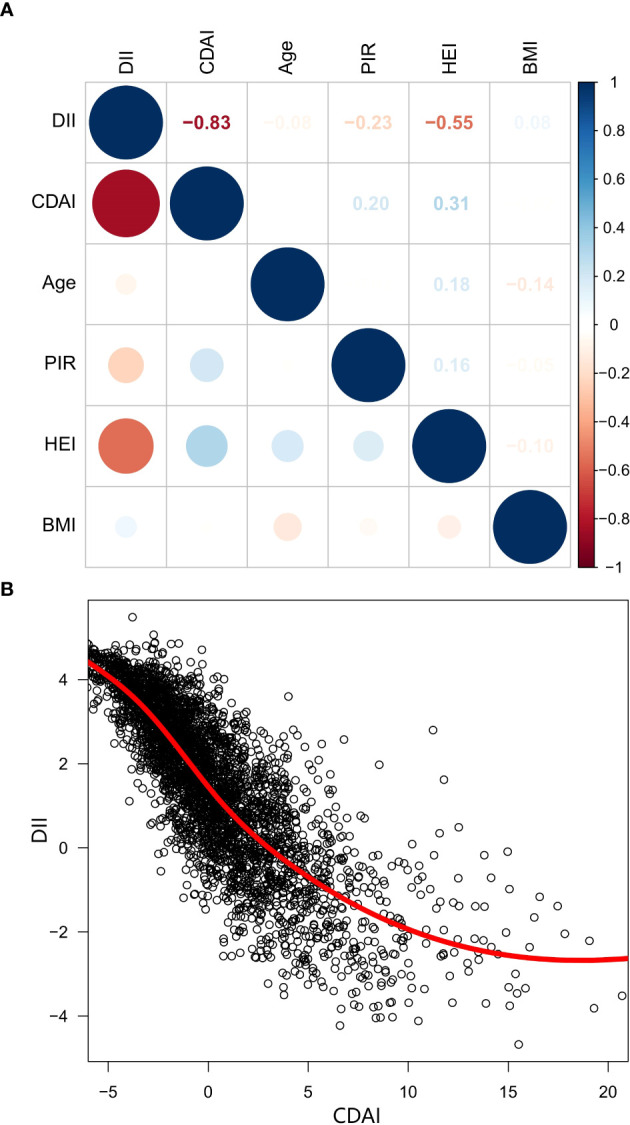
The correlation matrix and scatter plot of CDAI and DII. **(A)** Spearman’s correlation analysis, **(B)** Scatter plot with LOWESS fit. CDAI, composite dietary antioxidant index; DII, Dietary Inflammatory Index; PIR, Ratio of family income to poverty; BMI, body mass index; HEI, healthy eating index; LOWESS, Locally Weighted Scatterplot Smoothing.

To better understand the contrasting impacts of CDAI and DII on mortality risk in cancer survivors, we delved deeper into the connection between these two dietary indices. Our analysis using Spearman’s correlation method revealed a strong inverse relationship between CDAI and DII (r = -0.83) (**Figure 4A**), a finding that was also supported by the LOWESS fit curves (**Figure 4B**), which further illustrated the inverse association.

## Discussion

The expansion of cancer screening programs and advancements in treatment options have led to increased life expectancy and a growing number of cancer survivors all over the world. In the US, this population is projected to reach 22.1 million by 2030 (20), highlighting the need for innovative therapeutic approaches to enhance their survival state. In this context, this research investigation concentrated on individuals who suffered from cancers, firstly examined the impact of both DII and CDAI on the mortality risk of cancer survivors. The results revealed that a lower CDAI or higher DII was linked to a significantly increased risk of all-cause mortality, with these associations remaining significant after adjusting for various demographic and health factors, including age, sex and race, education level, PIR, BMI, HEI2015, marital status, smoking status, alcohol intake, diabetes, hypertension, and coronary heart disease. Moreover, our analysis revealed that this association holds true across various subgroups, lending credibility to the overall findings of the study. At last, an inverse correlation was observed between CDAI and DII in the diets of cancer survivors, providing insight into the contrasting effects of these two dietary indices on mortality risk.

Our research uncovered a significant positive association between the Dietary Inflammatory Index (DII) and overall mortality rates among cancer survivors, with the Kaplan-Meier survival curves and subgroup analyses confirming these results. These findings are consistent with those of a previous sub-analysis of the Iowa Women’s Health Study, which explored the link between diet-induced inflammation and mortality among older female cancer survivors, discovered that adhering to an anti-inflammatory diet and supplements could boost life expectancy in postmenopausal women who had survived cancers (21). Researches have consistently shown that long-term adherence to a Mediterranean-style diet and high scores on the Healthy Eating Index (HEI) is correlated with enhanced survival rates for cancer patients, due to the diet’s potent anti-inflammatory effects (22–24). Moreover, a diet rich in whole grains, vegetables, legumes, and fruits has been found to significantly lower the risk of death from all causes, primarily due to the high levels of anti-inflammatory compounds present in these foods (25–27). Conversely, various studies have revealed that diets characterized by a high inflammatory load are associated with a greater likelihood of developing cancers (28–30). The mechanism between a diet’s inflammatory potential and cancer-related mortality rates is not yet fully understood, but several possible explanations have been put forward. Consuming a diet that has the potential to trigger inflammation can activate pro-inflammatory molecules (14), which in turn can fuel the growth, survival, and migration of cancer cells, ultimately increasing the likelihood of cancer-related fatalities (31, 32). Such a diet may also lead to accelerated shortening of telomeres, a factor associated with a higher risk of death from all causes (33, 34). Furthermore, it is linked to higher inflammatory markers, including TNF-α, low-density lipoprotein, and low-density lipoprotein, all of which are tied to a greater risk of mortality (35, 36). Diets rich in saturated fats, which are often pro-inflammatory, have been shown to increase the risk of death from all causes, as well as from cancer and cardiovascular diseases (37). Given the crucial role of inflammation in the progression of tumors, it is probable that dietary factors impacts our vulnerability to disease and the likelihood of developing cancer by modifying the body’s inflammatory responses (38, 39), which supports with existing research findings.

A novel scoring system, known as CDAI, evaluates the total antioxidant value of an individual’s diet and can also reflect the body’s overall antioxidant capacity. Certain researchers propose that consuming antioxidants through diet may hinder tumor growth by counteracting free radicals and mending oxidative damage, thereby mitigating the harm caused by oxidative stress (40, 41). Conversely, some studies have found that taking antioxidant supplements may not improve patients’ survival and could even facilitate cancer spread (42). Our research confirms that a diet rich in antioxidants is associated with a lower risk of mortality from all causes among cancer survivors. Notably, the protective benefits of a high CDAI score are more pronounced in specific subgroups, including older males, former smokers or never smokers, individuals married/living with partner, and individuals with hypertension and diabetes. This finding is consistent with that observed by Song et al.’s study in colorectal cancer patients (25). Furthermore, a recent investigation revealed that consuming a diet rich in antioxidants can augment the effectiveness of immunotherapy in melanoma cases and lead to improved patient outcomes (13). Collectively, these findings imply that a diet high in antioxidants may lower mortality rates among cancer patients. The underlying mechanisms of this phenomenon involve the following aspects. Firstly, a diet rich in antioxidants is essential for enhancing the activity and function of immune cells, particularly those in the gut-associated lymphoid tissue (GALT). New research has revealed that a diet rich in antioxidants can encourage the growth of specific beneficial microorganisms in the gut, which in turn effectively regulate the composition of tumor-infiltrating mononuclear phagocytes (MPs) through the STING-IFN-I pathway. This can lead to a more favorable tumor microenvironment and increase the effectiveness of cancer treatments that rely on the immune system (43). Additionally, the antioxidant properties of dietary fiber breakdown products can stimulate the proliferation and anti-tumor response of CD8+ T cells by upregulating the expression of ID2, thereby amplifying the anti-tumor effect (44). Furthermore, chronic inflammation, a common characteristic of cancer patients, can weaken the anti-tumor ability of the body. However, consuming a diet abundant in foods high in antioxidants can help mitigate oxidative stress and exert an anti-inflammatory effect (45). By alleviating inflammation, it helps create a favorable immune environment, allowing the body to mount a more robust defense against cancer.

The investigation of the relationship between CDAI and DII scores showed an inverse correlation, indicating that these dietary factors tend to move in opposite directions, which may explain why DII and CDAI have different effects on individuals who suffered from cancer. This finding suggests that it may be feasible to simultaneously increase antioxidant intake and minimize pro-inflammatory components in one’s diet. Given that most foods contain a mix of pro-inflammatory and antioxidant properties, which are closely intertwined, a synergistic analysis was not conducted in this study. In brief, this study aimed to bridge a knowledge gap by examining the impact of CDAI and DII on cancer patient outcomes and exploring the connection between these two unique measures, thereby contributing to a deeper understanding of their prognostic value in cancer survivors. Notably, foods that contribute to a higher DII score include refined grains, red and processed meats, fried foods, sugary drinks, and high-fat dairy products (46, 47). Conversely, a diet rich in fruits, vegetables, and whole grains, such as the Mediterranean diet, which emphasizes low-fat and high-fiber intake, can help minimize dietary-induced inflammation in the body (48). A diet abundant in fiber and vitamins, featuring foods like legumes, fruits, and vegetables, may be particularly effective in combating oxidative stress (49). Our recommendation is that cancer patients make a conscious effort to incorporate more antioxidant-rich foods into their daily diet while limiting their consumption of pro-inflammatory foods.

This study also has several limitations that should be acknowledged. Firstly, the NHANES findings relied on self-reports from patients, potentially introducing recall bias. Self-reported dietary data is prone to recall bias and under reporting, particularly for unhealthy foods, which would result in nondifferential misclassification and be more likely to underestimate the true association towards the null result. Furthermore, the dietary data only reflected short-term dietary habits, making it difficult to examine how changes in diet over time relate to mortality. While 24-hour recalls provide valuable dietary information, they are susceptible to day-to-day variability and may not fully reflect habitual intake. Secondly, although the results were adjusted for various demographic and lifestyle factors, there may still be unknown confounding factors that influenced the results. Thirdly, The aggressiveness of tumors varies greatly, and as a result, patients undergo diverse treatment plans. Nevertheless, we did not categorize tumors because of the disparate number of tumors from different systems participating in this study. Fourthly, the diverse dietary patterns across different geographic locations and populations may impact the analysis of the relationship between CDAI and DII, as eating habits can significantly influence the results. Therefore, further prospective randomized controlled trials are necessary to validate these findings in the future.

## Conclusion

The research suggests that adopting a diet that low in pro-inflammatory foods and high in antioxidants may lower the all-cause mortality in cancer survivors. We hope that this research can provide valuable recommendations for enhancing cancer patients outcomes and offer insights for future clinical investigation.

## Data Availability

The original contributions presented in the study are included in the article/supplementary material. Further inquiries can be directed to the corresponding author.
